# Damage Effect of ALD-Al_2_O_3_ Based Metal-Oxide-Semiconductor Structures under Gamma-Ray Irradiation

**DOI:** 10.3390/mi12060661

**Published:** 2021-06-04

**Authors:** Man Ding

**Affiliations:** The College of Energy and Electrical Engineering, Hohai University, Nanjing 211100, China; ding.m@hhu.edu.cn; Tel.: +86-25-58099080

**Keywords:** aluminum oxide, radiation effect, charge trapping, defects

## Abstract

The radiation response of Al_2_O_3_ on silicon substrate under gamma-rays is studied in this article. The atomic layer deposited Al_2_O_3_ based metal-oxide-semiconductor structures were irradiated under gamma-ray with the total dose of 1.2 Mrad(Si)/2.5 Mrad(Si)/4 Mrad(Si). The generation, transportation and trapping characteristics of radiation induced charges were studied by using electronic, physical and chemical methods. Firstly, the radiation induced trapped charge density in Al_2_O_3_ is up to 10^12^ cm^−2^, with the effective trapping efficiency of 7–20% under irradiation. Secondly, the leakage current through Al_2_O_3_ changes little with the increase of radiation total dose. Thirdly, oxygen vacancy in Al_2_O_3_ and O dangling bonds and Al-Si metallic bonds at Al_2_O_3_/Si interface are dominant radiation induced defects in Al_2_O_3_/Si system, and the valence band offset between Al_2_O_3_ and Si is found to decrease after irradiation. From the results we can see that Al_2_O_3_ is radiation resistant from the aspect of leakage current and crystallization characteristics, but the radiation induced charge trapping and new defects in Al_2_O_3_/Si structure cannot be ignored. This paper provides a reference for the space application of Al_2_O_3_ based MOS devices.

## 1. Introduction

Microelectronic technology has been developing through the ‘Moore’s Law’ for several past decades and the feature size of the transistors in devices are reducing continuously. For now, the critical size of the transistors in advanced microelectronic devices has been shrinking to the point that the thickness of the gate dielectric of MOS(Metal-Oxide-Semiconductor) is comparable to several atoms size. The scaling of the microelectronic devices calls for new materials such as high-k materials for the gate dielectric and low-k materials for interconnecting technology. It also demands novel structures such as fin field-effect transistor, fully depleted silicon-on-insulator transistor and two-dimensional material devices and so on.

In advanced MOS devices, SiO_2_, which is widely used as the gate material in traditional MOS devices, is not applicable anymore as the leakage current and static power consumption would increase dramatically when the thickness of SiO_2_ decreases to lower than 2 nm, which would seriously impact the properties of MOS devices. Alternative materials with higher permittivity which can be called high-k materials have been proposed to replace SiO_2_ as the gate dielectric such as HfO_2_, Al_2_O_3_, ZrO_2_, La_2_O_3_ [1,2]. High-k materials that can be used as the gate dielectric in MOS devices must have a relatively larger bandgap to ensure large enough conduction band offset and valence band offset between the gate dielectric and the semiconductor substrate, which can restrict the charge transport and then reduce the leakage current of the device. Secondly, the high-k materials should be thermodynamically stable to avoid an interface layer forming from the chemical reaction on the dielectric/substrate interface. Thirdly, the high-k material should be electrically stable to prevent the generation of interface traps between the dielectric material and the substrate. Considering this, Al_2_O_3_ has been proposed as a promising candidate to replace SiO_2_ as the gate dielectric of MOS devices, with the relative dielectric constant around 8.9 and band gap around 8.7 eV that are both larger than that of SiO_2_. The higher dielectric constant ensures that the Al_2_O_3_ gate dielectric has large enough physical thickness to prevent charge tunneling through it. The larger band gap can guarantee that the conduction band offset and valence band offset between Al_2_O_3_ and the substrate are large enough to build sufficiently high electron and hole barriers on the interface to achieve relatively low leakage current and higher device performance.

Advanced MOS devices usually comprise different materials which are capable to withstand higher temperatures during technological treatment such as (HfO_2_)_x_(Al_2_O_3_)_(1−x)_/Si MOS structures. Al has been proposed to alloy HfO_2_ to raise the thermal stability and it was proven that the comprised gate dielectric with HfO_2_ and Al_2_O_3_ had higher crystallization temperature [3]. On the other hand, the resistance to oxygen diffusion of HfO_2_ was also improved by adding Al to form Hf aluminates, as Al_2_O_3_ has much lower oxygen diffusion coefficient than HfO_2_ at higher temperature [4].

Many researchers have studied the application of Al_2_O_3_ used as gate dielectrics in MOS devices. The deposition temperature and thermal annealing effects on the electrical characteristics of atomic layer deposited Al_2_O_3_ films on silicon were studied in [5]. The interface/border traps characterization in Al_2_O_3_/AlN/GaN structures were derived by dynamic capacitance dispersion technique in [6,7,8]. However, when the Al_2_O_3_ based MOS devices are working in aerospace, they would be damaged by the radiation environment in various forms such as the total dosage effect from gamma or X rays, displacement effect induced by heavy ions, and single event upset by high energy particles and so on. The total dosage effect from gamma or X rays significantly affect the operation of MOS devices by giving birth to a certain amount of charges inside the gate dielectric and then causing threshold voltage shift and leakage current increase. When Al_2_O_3_ based MOS devices are exposed to gamma or X rays, excessive electron-hole pairs would be generated and move inside Al_2_O_3_ and the charges would be trapped both in the dielectric bulk and on the interface between gate dielectric and silicon substrate forming the oxide and interface trapped charge [9,10]. On the other hand, the physical and chemical structures of the Al_2_O_3_ based MOS capacitors would also be changed when it is exposed to the radiation environment, including the crystallization characteristic and chemical bonding states change of the dielectric film. The electronic, physical and chemical transformation would degrade the Al_2_O_3_ based MOS devices and even result in the device failure. So, it is important to study the radiation response of the Al_2_O_3_ based MOS devices under gamma or X rays which would be useful for the radiation hardening of the microelectronic devices working in the space.

The radiation effect of Al_2_O_3_ based MOS devices has been studied by many research groups from the aspects of radiation induced charge trapping, trap characteristics and leakage current characteristics and so on. The charge trapping and passivation properties of Al_2_O_3_ based stack gate dielectric under gamma-ray irradiation was studied by using Capacitance-Voltage and Deep-Level Transient Spectroscopy measurements in [1,11,12]. The radiation induced defect characteristic as well as the leakage current in Al_2_O_3_ based metal-semiconductor-oxide structures under Si heavy ion were studied in [13]. The charge trapping property and charge transportation mechanism in Al_2_O_3_ were investigated and the origination of the leakage current and capacitance decrease were also obtained in this work. Except for gamma-ray and Si heavy ions, the radiation hardness of atomic layer deposited Al_2_O_3_ gate insulator in GaN-based MIS HEMTs (Metal-Insulator-Semiconductor High-Electron-Mobility Transistor) under proton irradiation was also studied in [14]. However, existing studies mostly focused on the electronic properties including the radiation induced charge trapping and leakage current characteristics, the failure mechanism of Al_2_O_3_-based MOS structures under irradiation were rarely studied in terms of physical and chemical characteristics which can provide a deeper understanding of radiation induced charge trapping and leakage current.

The total dose effect of Al_2_O_3_ based MOS structure under ^60^Co gamma-ray is studied in this paper. The Al_2_O_3_ thin films were fabricated on the p type silicon by using atomic layer deposition method and the radiation effect of the Al_2_O_3_ based MOS structures were studied in terms of electrical, physical and chemical characterization methods. Gamma-rays with the total dose of 1.2 M/2.5 M/4 Mrad(Si) were used as the radiation source. The radiation induced charge trapping characteristic was measured by high frequency capacitance-voltage (CV) method, from which the oxide and interface trapped charge densities in Al_2_O_3_/Si structure under different total dose were obtained. The effective trapping efficiency in Al_2_O_3_/Si structure was calculated from the threshold voltage shift after radiation. The leakage current characteristic was detected by using current-voltage (IV) measurement testing. The crystallization characteristic of the Al_2_O_3_ thin film was measured by using grazing incidence X-ray diffraction (GIXRD) method. The chemical bonding states and the band alignment of Al_2_O_3_/Si structure were studied by using X-ray photoelectron spectroscopy (XPS) and ultraviolet photoelectron spectroscopy (UPS) methods. The radiation response of the Al_2_O_3_ based MOS structure under gamma ray is summarized from the experimental results.

## 2. Sample Preparation and Characterization

Many technologies can be used to prepare Al_2_O_3_ thin films on silicon substrate, such as chemical vapor deposition method, sputtering, vacuum evaporation and atomic layer deposition methods. However, the ALD (Atomic Layer Deposition) technique has decisive advantage in high-k dielectric film manufacturing compared to the other methods, which can make full use of the surface saturation reaction and provides a high degree of stability. On the other hand, it’s easy to accurately control the dielectric film thickness by using ALD method, which is significant for very thin films with the thickness less than 10 nm.

In this paper, Al_2_O_3_ thin films were fabricated by using ALD method on p type silicon with the resistivity of 1–3 Ω·cm^−1^ and the crystal orientation of <100> by using TMA(Trimethylaluminium) and H_2_O as the precursor. Firstly, the silicon substrate was put into the reaction chamber after standard RCA (the standard semiconductor cleansing procedure initiated in the ‘RCA laboratory’ in N. J. Princeton) cleansing procedure. Secondly, TMA was then pushed into the reaction chamber by saturated vapor pressure and be adsorbed on the surface of the substrate, and redundant TMA was blow out by N_2_. Thirdly, H_2_O was brought into the chamber and reacted with TMA adsorbed on the substrate forming Al_2_O_3_, and then the impurities were blow out from the chamber by N_2_. The films were then annealed by rapid thermal annealing (RTA) at 450 °C for 60 s to strengthen the film cohesion and diminish the number of defects, and these Al_2_O_3_/Si structures were prepared for physical and chemical testing. For part of the chips with Al_2_O_3_/Si structures, aluminum was evaporated on the surface of Al_2_O_3_ films through a hard mask to form dot electrodes, and on the bottom of silicon substrate to form Al/Al_2_O_3_/Si structures for electronic measurement. These structures were then annealed by RTA process at 300 °C for 60 s to form ohmic contact between the electrode and the substrate. The thicknesses of the Al_2_O_3_ based MOS structures were 14.1 nm and 4.5 nm respectively, which were measured by using ellipsometer after deposition. Every chip with Al_2_O_3_/Si or Al/Al_2_O_3_/Si structures were divided into four equal parts and each part was irradiated by gamma-ray with different total doses.

^60^Co gamma-ray was used as the radiation source in this paper, and the total doses used in this article were 1.2 Mrad(Si)/2.5 Mrad(Si)/4 Mrad(Si) respectively with the dose rate of 100 rad(Si)/s, and all the samples were irradiated under gamma-ray with no voltage bias. The electronic, physical and chemical properties of more than eight numbers of Al/Al_2_O_3_/Si or Al_2_O_3_/Si samples were measured by using CV, IV, GIXRD, XPS and UPS methods before and after each total dose of gamma-ray irradiation. The radiation induced oxide and interface trapped charge density in Al_2_O_3_/Si system was calculated from the CV results by using Terman method, and the radiation induced leakage current was derived from IV characteristic. The chemical bonding states of Al_2_O_3_/Si system was extracted from XPS measurement and the radiation induced defect characteristic was then obtained. The valance band spectroscopy of Al_2_O_3_ was obtained from UPS measurement and the band offsets of Al_2_O_3_/Si was analyzed before and after radiation.

## 3. Experiment and Results

### 3.1. Crystallization Structure of Al_2_O_3_ Film before and after Irradiation

Crystallization characteristic of the gate dielectric film can significantly affect the transistor performance, as the grain boundaries between crystal lattices can act as the charge transport path which would raise the leakage current of the transistors. Considering this, researchers always prefer non-crystallized thin films to be the gate dielectric to ensure lower leakage current, but it is inevitable that the crystallization phases would be generated during post deposition thermal treatment and be changed in many other cases. Radiation rays with high energy might change the crystallization structure of the gate dielectric film, which can be detected by using XRD measurement.

**Figure 1:**
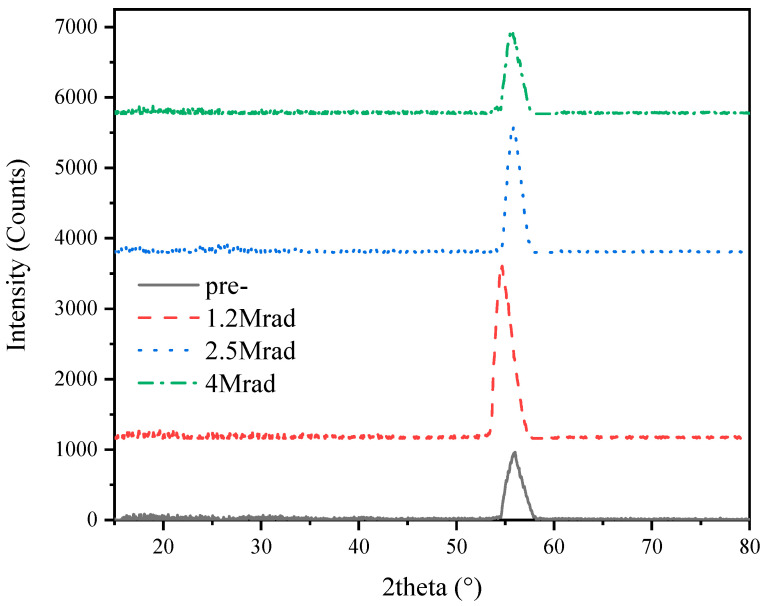
The grazing incidence X-ray diffraction (GIXRD) result of the Al_2_O_3_ thin film.

In view of the Al_2_O_3_ film thickness in this paper, GIXRD method was used to get the crystallization structure of the Al_2_O_3_ thin film on Si substrate with the grazing angle of 0.5° and scanning angle ranging from 15°~80°. The diffraction result is shown in Figure 1, illustrating that the diffraction peaks of the Al_2_O_3_ film before and after different total dose of irradiation were located around 55°, and the peak intensities of the irradiated Al_2_O_3_ films show no obvious change compared with that before irradiation. On the other hand, we can see that the Al_2_O_3_ thin film is mostly in amorphous state as the diffraction peak intensity shown on the spectrum is very low in value.

### 3.2. Chemical Structure and Band Alignment of Al_2_O_3_/Si Structure before and after Irradiation

#### 3.2.1. Chemical Structure of the Al_2_O_3_/Si Structure before and after Irradiation

**Figure 2:**
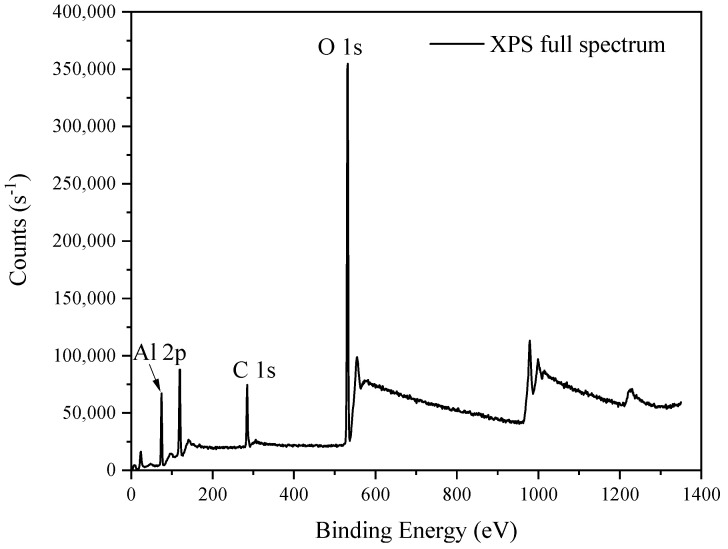
X-ray photoelectron spectroscopy (XPS) Full spectrum taken on the surface of Al_2_O_3_ thin film.

The performance of the Al_2_O_3_ based MOS devices can also be affected by the chemical structure including the constituent elements and the chemical bonding states between them, and new defects would be induced inside Al_2_O_3_ and on the interface between Al_2_O_3_ and Si after gamma-ray irradiation which can be measured by XPS method. The Al_2_O_3_-Si interface is the interface between Al_2_O_3_ film and the silicon substrate, which is in atomic scale with the value of a few tenths of nanometer. There would be weak Si-O bonds or some dangling bonds, which can introduce interface states to the band gap of Al_2_O_3_ on the interface. Ar^+^ etching was used to investigate the interface structure between 14.1 nm Al_2_O_3_ thin film and the silicon substrate for the reason that the penetration depth of X-ray of the equipment is limited to 3~5 nm which is smaller compared to the film thickness. The scanning binding energy in this paper ranged from 0 eV to 1400 eV and the full spectrum is shown in Figure 2. Every element including Al, Si, O has its’ unique binding energy on the XPS spectrum from which we can identify the chemical bonding state between the elements from the core levels on the spectrum. The binding energy peak at 284.8 eV on all the spectrums corresponds to the element carbon which might be introduced during MOS fabricating process or from the air exposure, so this core level of C 1s at 284.8 eV can be used as a reference of calibrate the equipment to eliminate the data deviation owing to the equipment’s charging effect.

**Figure 3:**
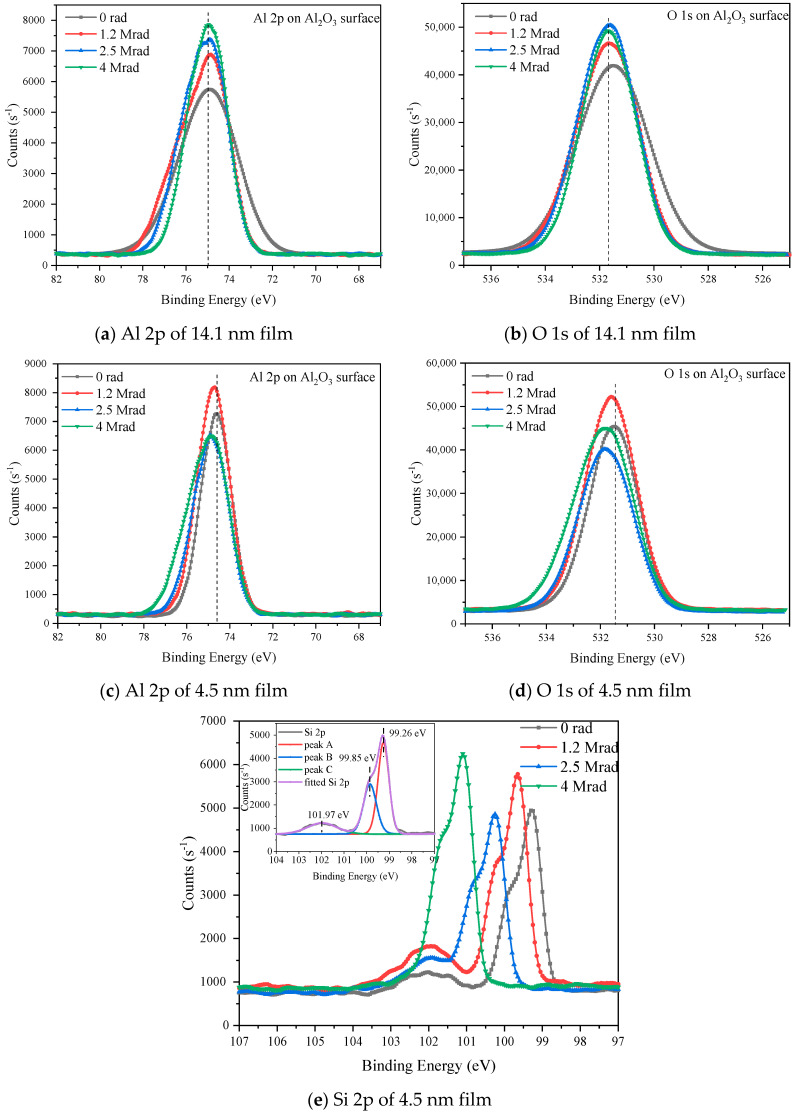
Detailed XPS results taken on the surface of Al_2_O_3_ film.

**Figure 4:**
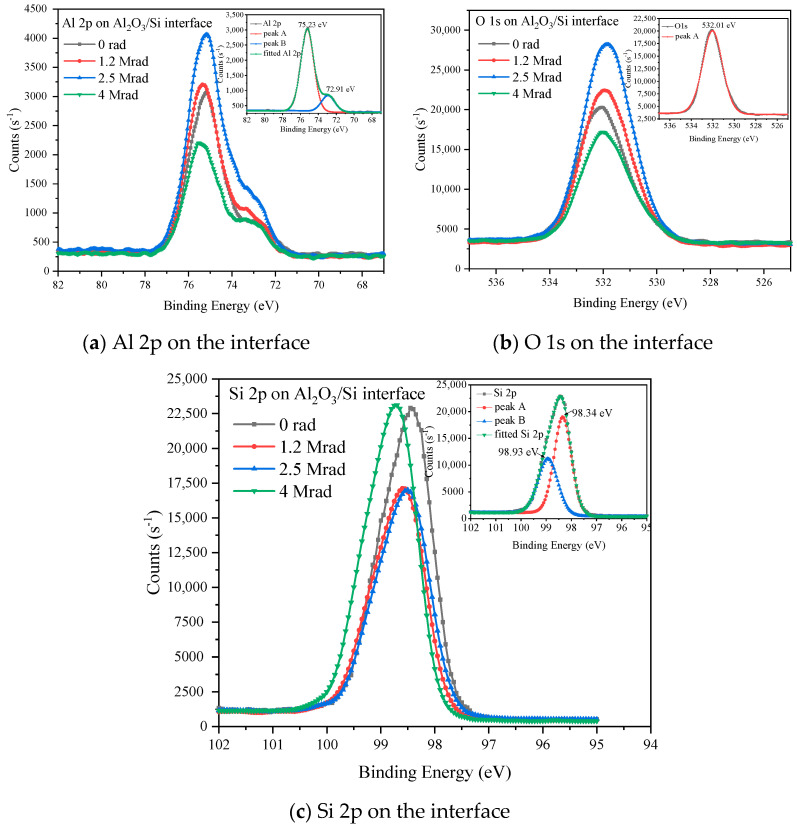
Detailed XPS results taken on Al_2_O_3_/Si interface.

We took the detailed XPS spectrum of Al 2p, O 1s, Si 2p to learn more about the chemical structure of Al_2_O_3_/Si structure and the results after data calibration by C 1s at 284.8 eV are shown in Figure 3. For the 14.1 nm Al_2_O_3_, the core levels of Al 2p, O 1s, and Si 2p taken from Al_2_O_3_ film surface are located at 74.9 eV, 531.5 eV, and 98 eV respectively, corresponding to the Al-O bonding and element silicon of the substrate. Moreover, the core levels of the elements show no obvious change with the increase of the radiation total dose implying that the chemical bonding states changed little and no new chemical bonding form was generated after gamma-ray irradiation with the total dose of up to 4 Mrad(Si). For the 4.5 nm Al_2_O_3_ film, the spectrum and core levels of Al 2p and O 1s are similar with that of the 14.1 nm Al_2_O_3_, and the change of the spectrums are not obvious too. However, the detailed XPS spectrum of Si 2p shows high peak intensity as the X-ray penetration depth is comparable to the film thickness. On the other hand, the Si 2p spectrum shows multi-peaks which are located at 99 eV to 102 eV, corresponding to the Si-O chemical bonding in SiO_x_. The detailed XPS spectrums of Al 2p, O 1s, Si 2p on Al_2_O_3_/Si interface are shown in Figure 4. What is noticeable in Figure 4 is that Al 2p and Si 2p have more than one peak in their detailed scans. This indicates that there is more than one valence state for Al and Si, as every valence state of the chemical elements has its’ unique core level on the XPS spectrum according to the principle of XPS measurement. In order to specify the valence states, multi-peak resolution was applied, and results are shown in the insets of Figure 4. Peak A and peak B correspond to the core levels of each chemical bonding form for the elements. The core levels of each element are located around 75 eV and 73 eV for Al 2p, 532 eV for O 1s, and 98.9 eV and 98.3 eV for Si 2p, implying that there are Al-O bonds, and Al-Si metallic bonds on the interface between Al_2_O_3_ and Si substrate.

Semiquantitative method was applied to figure out the atomic concentration ratio of elements by using Equation (1), and the atom ratios between Al and O elements of Al_2_O_3_ thin film before and after radiation are shown in Table 1.

(1) $$\frac{n_{Al}}{n_{O}}=\frac{I_{Al2p}/S_{Al}}{I_{O1s}/S_{O}}$$

where, *n_Al_/n_O_* is the atomic concentration ratio between *A*l and O, *I_Al_*_2*p*_ and *I_O_*_1*s*_ are the intensity of the binding energy peaks of *Al*2*p* and *O*1*s* at XPS detailed spectrum in the chemical bonding form of Al_2_O_3_, *S_Al_* and *S_O_* are the sensitive factors of each element with the value of 0.56 and 2.881 determined by the test equipment we used in this paper.

For 14.1 nm Al_2_O_3_, the atomic ratio between Al and O inside Al_2_O_3_ thin film is around 0.7 which is close to but a little larger than the standard value 0.66. This indicates that the Al_2_O_3_ in this paper is basically stoichiometric which is fabricated by ALD method and there are oxygen vacancies inside the Al_2_O_3_ film bulk with the amount increase with the increase of radiation total dose. The Al/O atomic ratio on (14.1 nm) Al_2_O_3_/Si interface is 0.66 before radiation and gets lower and lower with the increase of the radiation total dose, implying that the interface is also stoichiometric and Al-O bonds were broken after irradiation leaving O dangling bonds and Al-Si metallic bonds on the interface, which is confirmed by the detailed XPS result of Al 2p and Si 2p on the interface. For the 4.5 nm Al_2_O_3_, it was found that there are Al-O and Si-O bonds in 4.5 nm Al_2_O_3_ film from Figure 3c–e, but it is difficult to differentiate the O 1s peak as the binding energies of Al-O and Si-O are very close to each other. We used the semiquantitative method to roughly estimate the Al/O and ratio as shown in Table 1, and it is found that he Al/O atomic ratio is close to but a little smaller than the stoichiometric value 0.666, which might be resulted from the existence of O 1s from Si-O. The Al/O ratio changes very little after radiation. We also roughly estimated the Si/O atomic ratio from the detailed XPS results by applying the sensitive factor of 0.9 for Si, and they are 0.156, 0.176, 0.186, and 0.209 before and after each total dose of irradiation. Although the calculated Si/O ratio is not accurate as we applied the whole O 1s peak, the relative change of the Si/O ratio after each total dose of irradiation does make sense. It shows that the Si/O ratio increases with the increase of radiation total dose, indicating that oxygen vacancies were generated after irradiation. In summary, gamma-ray irradiation would introduce oxygen vacancies and dangling bonds to the Al_2_O_3_/Si structure. The MOS structures would degrade by the raise of the oxide and interface trapped charge density.

#### 3.2.2. Band Alignment of Al_2_O_3_/Si Structure under Radiation

**Figure 5:**
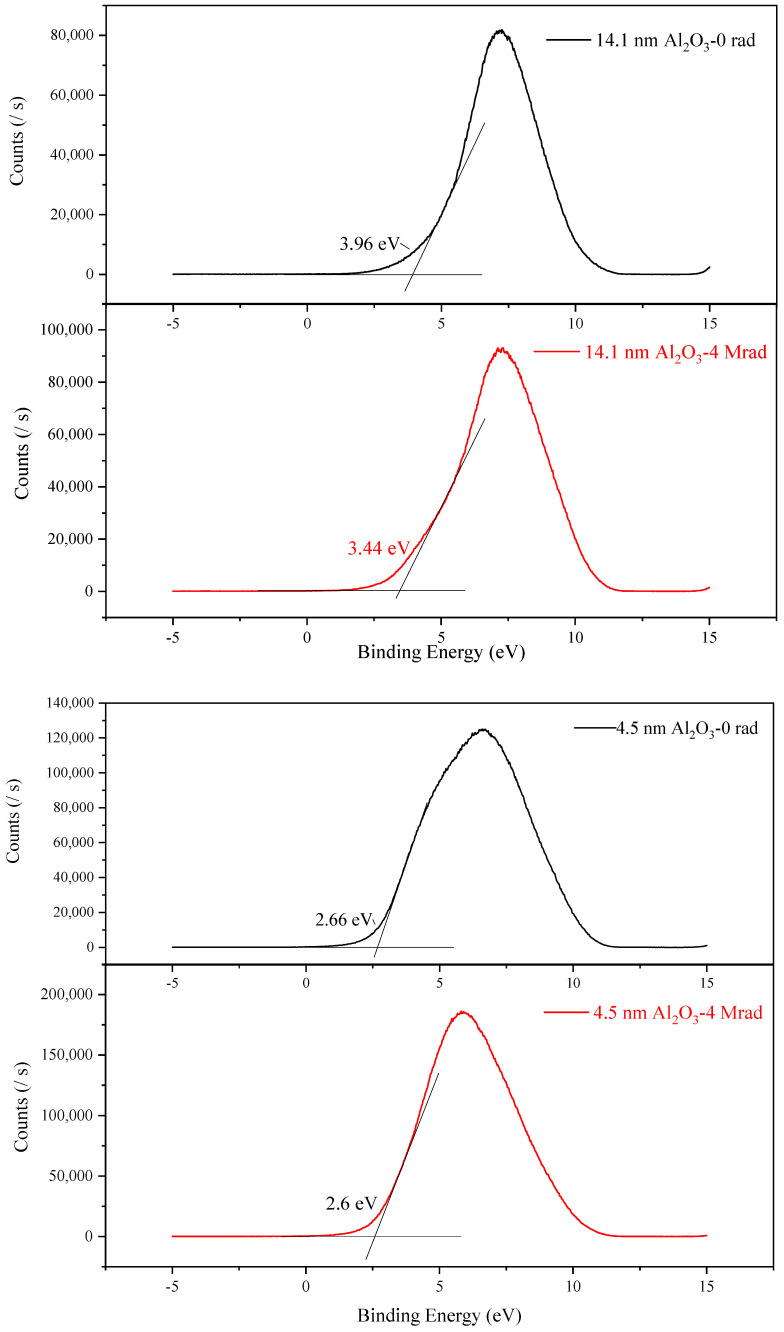
Valence band spectrum of Al_2_O_3_/Si structure taken by ultraviolet photoelectron spectroscopy (UPS).

The band structure of the Al_2_O_3_/Si significantly affect the interface property of the MOS structure as the valence band and conductance band offsets significantly influence the charge transportation characteristic which can be measured by UPS method. The valence band spectrums of Al_2_O_3_/Si structures are shown in Figure 5. The valence band offset between Al_2_O_3_ and Si substrate on the interface can be figured out by Equation (2).

(2) $$\Delta E_{v}=E_{VBM}^{Al_{2}O_{3}}-E_{VBM}^{Si}$$

where, Δ*Ev* is the valence band offset between *Al*_2_*O*_3_ and *Si* substrate, $E_{VBM}^{Al_{2}O_{3}}$ is the valence band maximum of *Al*_2_*O*_3_ film which can be distinguished from the valence band spectrum of *Al*_2_*O*_3_ by using extrapolation method, and $E_{VBM}^{Si}$ is the valence band maximum of *Si* substrate.

The valence band maximums before and after 4 Mrad(Si) irradiation figured out by using extrapolation method are 3.96 eV and 3.44 eV for 14.1 nm Al_2_O_3_ and are 2.66 eV and 2.6 eV for 4.5 nm Al_2_O_3_, respectively. The empirical value of valence band maximum of Si is 0.1 eV with which we can calculate the valence band offset between Al_2_O_3_ and Si. From Equation (2), the valence band offsets at Al_2_O_3_/Si interface before and after 4 Mrad(Si) irradiation are 3.86 eV and 3.34 eV for 14.1 nm Al_2_O_3_, and are 2.56 eV and 2.5 eV for 4.5 nm Al_2_O_3_. We can see that the valence band offset at Al_2_O_3_/Si interface decreases after irradiation, which is resulted from the newly formed interface traps induced by radiation. From XPS results of Al_2_O_3_/Si structure we have known that gamma-ray irradiation would introduce interface traps to the structure which is confirmed by the valence band offset decrease in this part. What’s noticeable is that the valence band offset at Al_2_O_3_/Si interface for the 4.5 nm Al_2_O_3_ changes very little after 4 Mrad(Si) irradiation, implying that the thinner Al_2_O_3_ film would be more radiation resistant compared to the thicker films from the aspect of band alignment, which is in accordance with the XPS results.

### 3.3. Radiation Induced Charge Trapping and Transportation in Al_2_O_3_ Based MOS Structures

The total dose effect of gate dielectric is originated from the generation of large number of electron-holes pairs owing to the ionization induced by gamma-ray irradiation. Part of these electron-hole pairs would first compound with each other and most of the left electrons would be swept out of the dielectric layer, leaving a certain number of radiation induced charges inside the gate dielectric. The charges would be trapped in the MOS structure forming oxide and interface trapped charges and give rise to radiation induced leakage current. The oxide trapped charges are located inside the bulk of Al_2_O_3_ film, and the interface trapped charges are located in Al_2_O_3_ near the Al_2_O_3_/Si interface within the range of a few tenths of nanometer from the silicon substrate. In this part, the radiation induced charge trapping characteristic and the leakage current is studied.

**Figure 6:**
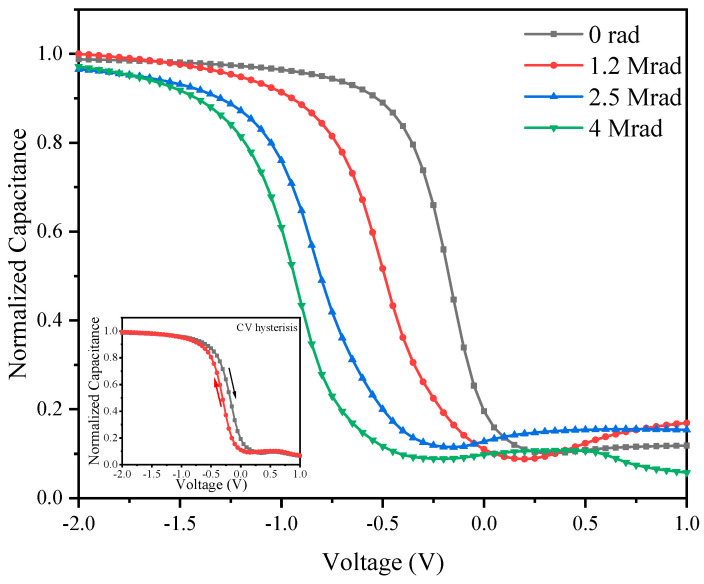
CV characteristic of the 14.1 nm Al_2_O_3_ based MOS structures before and after irradiation.

High frequency CV measurement was taken on the Al/Al_2_O_3_/Si MOS structure with the scanning voltage ranging from −2 V- + 1 V by the voltage interval of 0.05 V. The accumulation area capacitance in the CV curve is 1.4847 × 10^−9^ F, from which the relative dielectric constant can be figured out to be 8.368, which is very close to the empirical relative dielectric constant of Al_2_O_3_, indicating that the thin gate dielectric film prepared by ALD method has good thermal and electrical performance. The normalized CV curve of 14.1 nm and 4.5 nm Al_2_O_3_ film based MOS structures are shown in Figure 6, with the inset showing the CV hysteresis curve.

**Figure 7:**
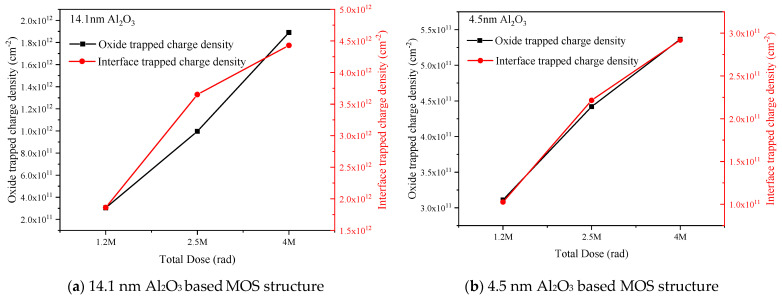
Oxide and interface trapped charge density in Al_2_O_3_ based MOS structures.

The CV curves of the MOS structures shift to the negative voltage direction after radiation and the voltage shift increases with the increase of the radiation total dose, indicating that positive oxide trapped charge were generated inside Al_2_O_3_ gate dielectric during gamma-ray irradiation. On the other hand, the post-radiation CV curves show stretch-out which demonstrates the generation of interface trapped charge in the MOS structure. The radiation induced oxide and interface trapped charge densities can be figured out by using Terman method and the results are shown in Figure 7, and the results are average value of more than eight samples which have very low CV hysteresis.

For 14.1 nm Al_2_O_3_, the oxide trapped charge densities are in the order of 10^11^–10^12^ cm^−2^, which increase with the increase of radiation total dose. The interface trapped charge densities are in the order to 10^12^ cm^−2^, which also increase with the increase of radiation total dose. For 4.5 nm Al_2_O_3_, the oxide trapped densities are in the order of 10^11^ cm^−2^, and the interface trapped charge densities are in the order of 10^11^ cm^−2^, and both of them increases with the increase of radiation total dose. Higher radiation total dose results in larger number of electron-hole pairs and larger number of oxide and interface trapped charge, which is confirmed by the results shown in Figure 7. Moreover, the radiation induced oxide trapped charge density in 4.5 nm Al_2_O_3_ is much less than that in 14.1 nm Al_2_O_3_ under 4 Mrad(Si) irradiation, which might be resulted from the lower number of intrinsic and lower number of radiation induced defects that can act as the trapping centers. It has been proved by the XPS results in 3.2.1, in which the atomic ratio between Al and O of 4.5 nm Al_2_O_3_ is closer to 0.66 than that of 14.1 nm Al_2_O_3_ implying that the number of oxygen vacancies is smaller in the thinner Al_2_O_3_ film. On the other hand, the change of the atomic ratio is lower in 4.5 nm Al_2_O_3_ than that in 14.1 nm Al_2_O_3_ after 4 Mrad(Si) gamma-ray irradiation. Moreover, the radiation induced interface trapped charge density in 4.5 nm film is one order less than that in 14.1 nm film under each total dose of irradiation, this is in accordance with the Al-O atomic ratio change in the XPS result.

Trapping efficiency is a dimensionless quantity used to approximate the intrinsic “trappiness” of the insulator. The effective trapping efficiency of an alternative dielectric is defined as what the trapping efficiency would be if the gate dielectric were SiO_2_ instead of an alternative dielectric. This definition is consistent with the concept of EOT, which describes what the thickness of the dielectric would be if it were SiO_2_ instead of an alternative dielectric, based on the measured capacitance value [15,16]. As for the effective trapping efficiency in the ALD Al_2_O_3_ based MOS structure, it can be carried out by using Equation (3).

(3) $$f_{ot}=-\frac{\Delta V_{mg}\varepsilon_{ox}}{q\kappa_{g}f_{y}t_{eq}t_{phys}D}$$

where, *f_ot_* is the effective trapping efficiency in Al_2_O_3_, Δ*V_mg_* is the radiation induced midgag shift, *ε_ox_* is the permittivity of SiO_2_, *q* is electronic charge, *κ_g_* is the electron-hole pair generation per unit dose in Al_2_O_3_ which is the known value of charge generation in SiO_2_(~8.1 × 10^12^ cm^−3^·rad^−1^ in [17]) scaled by the ratio of the bandgap of SiO_2_ to the bandgap of Al_2_O_3_ [15], *f_y_* is the charge yield in SiO_2_ in the same electric field, i it was calculated by the method in [18,19] based on the electric field and the radiation source (~0.5), *t_eq_* is the equivalent oxide thickness, *t_pyhs_* is the physical thickness of Al_2_O_3_, *D* is the radiation total dose.

The effective trapping efficiencies in 14.1 nm Al_2_O_3_ are 7.7%, 8.5% and 10.7% under 1.2 Mrad(Si) (approximately 0.69 Mrad(SiO_2_)), 2.5 Mrad(Si)(approximately 1.45 Mrad(SiO_2_)) and 4 Mrad(Si)(approximately 2.32 Mrad(SiO_2_)), respectively. The effective trapping efficiencies in 4.5 nm Al_2_O_3_ are 19.6%, 13.4% and 10.2% under 1.2 Mrad(Si), 2.5 Mrad(Si) and 4 Mrad(Si) irradiation, respectively. The trapping efficiencies in ALD Al_2_O_3_ in this paper are lower compared with the effective trapping efficiency in SiO_2_ reported in [15] ranging from a few percent up to 50%, which is dependent primarily on the number of oxygen vacancies in the oxide. This result shows that the radiation induced charge trapping in ALD-Al_2_O_3_ is not critical as that in as-deposited SiO_2_, but it is still larger than that in radiation hardened SiO_2_ in [15]. So, the radiation hardness of ALD Al_2_O_3_ based MOS devices still needs further research before it is used in space environment.

The leakage current through the Al_2_O_3_ based MOS structure is very important parameter reflecting the device performance, and the leakage current characteristic of the MOS structures were measured by using Keithley 4200 SCS with the scanning voltage ranging from −2 V- + 3 V by the voltage interval of 0.05 V. The leakage current is around 3 × 10^−7^ A at + 1 V which changes very little after irradiation, indicating that the radiation induced leakage current is not the key factor of the damage effect of the Al_2_O_3_ based MOS structures.

## 4. Discussion

The radiation damage effect of Al_2_O_3_ based MOS structures is studied in this paper from the aspects of electronic, physical and chemical properties. From the physical structure results measured by GIXRD method, we have found that the diffraction peak of the Al_2_O_3_ film located at around 55°on the diffraction spectrum and the diffraction peaks are not so sharp with relatively low peak intensity, indicating that the Al_2_O_3_ film is dominated by amorphous states. The deposition temperature and post-annealing temperature was not high enough to form perfect crystallization phases which depends strongly on the thermal treatment process. On the other hand, Al_2_O_3_ films grown directly on Si was reported to remain amorphous up to 1000 °C [4]. These are the reasons of the amorphous state of the ALD Al_2_O_3_ dielectric film that can produce excellent electrical characteristics. Moreover, the peak location as well as the peak intensity change little after irradiation, implying that the amorphous dominated Al_2_O_3_ film is radiation resistant as the crystallization changes little after radiation. Furthermore, research has reported that doping HfO_2_ by Al raises the film crystallization temperature of HfO_2_ and thus drastically reduces the oxygen diffusion along the grain boundaries during annealing in [4,20]. In this paper, the leakage current through Al_2_O_3_ also changes little after irradiation, which is in accordance with the crystallization characteristic as the grain boundaries are always thought to be charge transport channels. The impact of gamma radiation on charge trapping properties of nanolaminated HfO_2_/Al_2_O_3_ ALD stacks was studied in [12], and no leakage deterioration was detected for the investigated stacks and doses which supports the result in this paper.

The radiation induced damage is dominated by the newly formed defects and the charge trapping [21]. From the XPS results, we can use ALD method to fabricate a stoichiometric Al_2_O_3_ film with low density of intrinsic defect both inside the oxide and on Al_2_O_3_/Si interface. We have found that oxygen vacancy is dominant oxide defects inside Al_2_O_3_ film, which increase with the increase of irradiation total dose. The Al_2_O_3_/Si interface defects are dominated by O dangling bonds and Al-Si metallic bonds, which also increases with the increase of irradiation total dose. The Al-O bonds would have been broken under the high energy of gamma-ray irradiation, leaving new oxygen vacancies inside Al_2_O_3_ film and leaving dangling bonds and Al-Si metallic bonds at Al_2_O_3_/Si interface, which would act as the trapping centers in the MOS structures. Moreover, weak Si-O bonds were found in the thinner film, indicating that there might be oxygen penetration in Al_2_O_3_ film. Oxygen would penetrate to the Al_2_O_3_/Si interface and react with silicon substrate, forming weak Si-O bonds. These weak Si-O bonds would also participate in the irradiation damage effect of the film. Metallic bonds on the gate dielectric/substrate interface would be induced from the combine of Al and Si on the interface resulting from the oxygen diffusion to Si substrate [22]. On the other hand, researchers have proposed that H^+^ transportation plays an important role in the radiation induced interface defect formation between gate dielectric and silicon substrate, the passivated Si dangling bonds by H would be broken after radiation and form Si dangling bonds [23,24], and this might facilitate the formation of Al-Si metallic bonds on the interface. The interface defects would also affect the band offsets between Al_2_O_3_ and Si substrate, which is confirmed by the UPS results that the valence band offset decreases after 4 Mrad(Si) irradiation.

According to the total dosage effect theory, radiation would give rise to large number of electron-hole pairs which would be trapped and move inside the MOS structure after initial compound. The trapped charge density in Al_2_O_3_ based MOS structures is dependent not only on the charge yield but also on the effective trapping efficiency and the new trap generation after radiation. The effective trapping efficiencies are in the range of 7% to 20% in the 14.1 nm and 4.5 nm Al_2_O_3_ film, which are lower compared with that in as- deposited SiO_2_ but still larger than that in radiation hardened SiO_2_ [15]. The oxide trapped charge in Al_2_O_3_ is positive which increases with the increase of irradiation total dose, and the interface trapped charge at Al_2_O_3_/Si interface is also positive and also increase with the total dose. The oxide and interface trapped charge density are in the order of 10^11^ cm^−2^–10^12^ cm^−2^ in 14.1 nm Al_2_O_3_ based MOS structure and are in the order of 10^11^ cm^−2^ in 4.5 nm Al_2_O_3_ based MOS structure, this result supports [5,11]. As the effective trapping efficiency is similar, the lower radiation induced trapped charge density in thinner Al_2_O_3_ film might be resulted from the lower defect density as shown in the XPS results. Moreover, as the interface characteristic between Al_2_O_3_ gate dielectric and the substrate would influence the performance of the MOS structure, we can introduce an interface layer to the MOS structure. It was reported that the interface characterization was improved after adding an AlN interface between Al_2_O_3_ and the substrate in [6].

In summary, the radiation damage in Al_2_O_3_ based MOS structure is dominated by charge trapping and trap generation, and the radiation hardness of Al_2_O_3_ film needs further research before it is used in space environment.

## 5. Conclusions

The radiation response of ALD Al_2_O_3_ based MOS structures were studied in this article, and we can conclude the radiation response as following:

(1) The radiation induced oxide and interface trapped charges are positive in the order of 10^11^ cm^−2^–10^12^ cm^−2^ in 14.1 nm Al_2_O_3_ and 10^11^ cm^−2^ in 4.5 nm Al_2_O_3_, which increase with the radiation total dose.
(2) The radiation induced defects are oxygen vacancies in Al_2_O_3_ bulk and O dangling bonds and Al-Si metallic bonds on Al_2_O_3_/Si interface, which increase with the increase of total dose. The effective trapping efficiencies in Al_2_O_3_ film are in the range of 7% to 20% under each total dose of gamma-ray irradiation.
(3) The physical structure of Al_2_O_3_ shows no obvious change after radiation, and the leakage current through Al_2_O_3_ film also changes little after radiation which is in accordance with the physical characteristic.

In summary, ALD method is a favorable fabricating method for high-k gate dielectrics as the Al_2_O_3_ film is basically stoichiometric and the film is almost amorphous. The radiation induced charge trapping and radiation induced defect generation are responsible for the damage effect of Al_2_O_3_ based MOS structures. The radiation induced trapped charge densities are not very large, but the radiation induced defects would prevent the use of Al_2_O_3_ based MOS devices in space environment. Considering this, the ALD Al_2_O_3_ should be radiation hardened, such as introducing a SiO_2_ layer to form stacked gate dielectric layer to improve the electrical and chemical characteristic on the interface or doping some other elements to restraint the defect generation. All in all, the ALD Al_2_O_3_ film is radiation resistant to some extent which makes it a promising candidate of new high-k gate dielectrics for MOS devices working in space. Before that, the radiation hardness of Al2O3 based MOS devices needs further research.

## Figures and Tables

**Table 1:** Atomic concentration ratio between Al and O in the Al_2_O_3_/Si structure.

Total Dose	14.1 nm Al_2_O_3_ (Surface)	14.1 nm Al_2_O_3_ (Interface)	4.5 nm Al_2_O_3_
Pre-	0.718	0.66	0.656
1.2Mrad	0.725	0.61	0.657
2.5Mrad	0.739	0.61	0.661
4Mrad	0.754	0.56	0.661

## Data Availability

The data presented in this study are available on request from the corresponding author. The data are not publicly available due to privacy reason.
